# The role of telomere-binding modulators in pluripotent stem cells

**DOI:** 10.1007/s13238-019-0651-y

**Published:** 2019-07-27

**Authors:** Feng Li, Yuanlong Ge, Dan Liu, Zhou Songyang

**Affiliations:** 1 State Key Laboratory of Oncology in South China, Cancer Center, Collaborative Innovation Center for Cancer Medicine, and Key Laboratory of Gene Engineering of the Ministry of Education, School of Life Sciences, Sun Yat-sen University, Guangzhou, 510275 China; 2 Verna and Marrs Department of Biochemistry and Molecular Biology, Baylor College of Medicine, One Baylor Plaza, Houston, TX 77030 USA

**Keywords:** telomere, pluripotent stem cells, telomerase, ALT, shelterin/telosome complex

## Abstract

Pluripotent stem cells (PSCs) such as embryonic stem cells (ESCs), ESCs derived by somatic cell nuclear transfer (ntESCs), and induced pluripotent stem cells (iPSCs) have unlimited capacity for self-renewal and pluripotency and can give rise to all types of somatic cells. In order to maintain their self-renewal and pluripotency, PSCs need to preserve their telomere length and homeostasis. In recent years, increasing studies have shown that telomere reprogramming is essential for stem cell pluripotency maintenance and its induced pluripotency process. Telomere-associated proteins are not only required for telomere maintenance in both stem cells, their extra-telomeric functions have also been found to be critical as well. Here, we will discuss how telomeres and telomere-associated factors participate and regulate the maintenance of stem cell pluripotency.

## TELOMERES

Telomeres are ends of linear chromosomes that are bound by a host of protein factors and form a complex nucleoprotein structure. In mammalian cells, telomeres are consisted of repetitive [TTAGGG]n sequences and protected by the core six-protein shelterin/telosome complex (Xin et al., 2008; de Lange, 2018). The shelterin/telosome complex in turn recruits many other factors from diverse cellular pathways to telomeres for protection and maintenance. The telomeric DNA adopts the protective T-loop structure where the single-stranded 3′ overhang invades into the double-stranded telomeric region (Griffith et al., 1999; Doksani et al., 2013), and thus protected from nucleolytic degradation and activation of the DNA damage response. Due to the end replication problem, telomeres undergo gradual shortening during proliferation in normal somatic cells. Telomere attrition during successive cell divisions can ultimately lead to chromosomal instability and contribute significantly to genomic rearrangements that can result in tumorigenesis.

## TELOMERES IN PSCs

**Figure 1:**
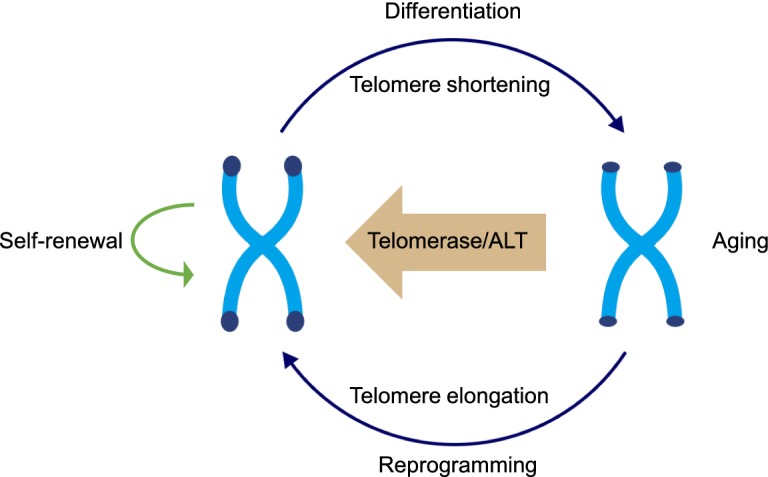
**Telomere length and pluripotency**. Due to the end replication problem, telomere shortening occurs in stem cell differentiation and aging. Through telomerase and the ALT mechanism, telomeres are elongated to sustain self-renewal

Pluripotent stem cells (PSCs), including embryonic stem cells (ESCs), nuclear transfer ESCs (ntESCs) and induced pluripotent stem cells (iPSCs), have the potential of unlimited-proliferation and the capacity to differentiate into any cell type. Therefore, PSCs can be used for any cell type replacement and have great value for regenerative medical. Proper regulation of telomere length and telomere-binding proteins is critical for telomere function and genomic stability, both in somatic cells and PSCs. Mouse ESCs and/or iPSCs with short telomeres exhibited unsuccessful teratoma formation and chimera production and failed to produce ES/iPS mice (Huang et al., 2011). It has been reported that reprogramming by retroviral transduction of iPS factors of mouse primary embryonic fibroblasts also resulted in a dramatic increase in telomere length, similar to what was observed in ES cells (Marion et al., 2009). Furthermore, telomeres were also elongated during reprogramming of human blastocysts to embryonic stem cells and reached a relatively stable level in these iPSCs (Zeng et al., 2014). Cells with longer telomeres may have higher pluripotency potential and be positively selected from a heterogeneous population. Single-cell telomere length analysis coupled with RNA sequencing (scT&R-seq) revealed that hESCs with short telomeres exhibited more characteristics associated with differentiated lineages, whereas those with longer telomeres better maintained pluripotency (Wang et al., 2017). Telomere length also plays an important role in stem cell differentiation. iPSCs with relatively long telomeres were found to differentiate more to the mesoderm and endoderm lineages, such as cardiac progenitor cells (Aguado et al., 2017). In summary, telomere length may help determine the pluripotency status of stem cells (Fig. 1).

How the state of telomere affects stem cell pluripotency is an essential question. Previous studies have shown that the telomeric chromatin appears more open in PSCs than in somatic cells, and becomes more repressive during differentiation (Marion et al., 2009; Wong et al., 2009). Conversely, the reprogramming of adult somatic cells into pluripotent stem cells can “switch” the telomeric chromatin from a repressive state to more open and stem cell-like. Although how such telomeric chromatin changes affect stem cell pluripotency remains unknown, it is clear that telomeric chromatin dynamics has a fundamental role in ensuring stem cell pluripotency.

## TELOMERE MAINTENANCE IN PSCS

One of the hallmarks of stem cells is the presence of unlimited growth potential, which is enabled by a telomere maintenance mechanism (TMM) capable of completely counteracting normal telomere shortening. PSCs can proliferate indefinitely while maintaining pluripotency and avoid telomere attrition and genome instability by elongating and protecting their telomeres. Short telomeres in ESCs lead to reduced pluripotency and unstable differentiation (Pucci et al., 2013). As is the case for somatic cells, two mechanisms exist to ensure telomere lengthening in ESCs, elevating telomerase activity and alternative lengthening of telomeres (ALT) (Liu et al., 2007). Losing telomerase activity in ES/iPS cells was shown to result in critically shortened telomeres (Huang et al., 2011; Pucci et al., 2013), supporting a major role for telomerase in telomere maintenance in PSCs.

The telomerase holoenzyme adds telomeric DNA repeats to the ends of chromosomes (Greider and Blackburn, 1989; Lingner et al., 1997). It is a specialized ribonucleoprotein complex, composed of the reverse transcriptase TERT and the RNA template TERC as well as associating proteins such as the ribonucleoprotein dyskerin DKC1 (Cohen et al., 2007). Unlike normal somatic cells, telomerase is usually activated and expressed in stem and cancer cells. Telomerase-dependent telomere maintenance is required for pluripotency and long-term self-renewal of human and mouse PSCs (Huang et al., 2011; Huang et al., 2014; Liu, 2017).

Despite the major role telomerase plays in ESC establishment and iPSC generation, mouse TERC^−/−^ and TERT^−/−^ ESCs survivor lines were found to maintain their telomere length using the telomerase-independent mechanism ALT (Niida et al., 2000; Wang et al., 2005). The ALT pathway has been found in ~10%–15% cancers and is usually characterized by the presence of ALT-associated promyelocytic leukemia (PML) bodies (APBs), extrachromosomal telomeric repeats (ECTR), and high frequency of telomere sister chromatid exchange (T-SCE) in cells (Cesare and Reddel, 2010). Interestingly, mESCs and early cleavage stage embryos might have elongated their telomeres using an ALT-like mechanism, displaying rapid telomere elongation along with increased T-SCE (Liu et al., 2007; Zalzman et al., 2010). Telomere maintenance by telomerase and the ALT pathway may coexist in human cells under certain circumstances in cancer cells (Cerone et al., 2001), and in ESCs and iPSCs, as was reported in studies that found telomerase reactivation as well as ALT characteristics in these cells (Wang et al., 2012; Chang et al., 2013). More work is required for a true understanding of the mechanisms of ALT pathway activation and utilization in pluripotency maintenance.

## TELOMERASE IN PSCS

Although the telomerase itself is dispensable for establishing ESC or iPSC lines (Marion et al., 2009; Huang et al., 2011; Wang et al., 2012), telomerase-deficient mice are viable for only six generations (Huang et al., 2014). TERC^−/−^ mESCs with short telomeres showed genome-wide DNA hypomethylation and altered H3K27me3 modification, which led to unstable ESC differentiation *in vitro* (Pucci et al., 2013). TERT knockout hESCs showed progressive telomere shortening, which resulted in apoptosis and/or limited proliferation (Liu et al., 2016). Further, TERC^+/−^ mESCs had a significantly reduced ability to generate ESC mice compared with wild-type mESCs (Huang et al., 2011). And late-generation TERC^−/−^ mice often exhibit striking phenotypes associated with telomere dysfunction, including chromosomal abnormalities, development defects, aging and tumor formation (Blasco et al., 1997; Herrera et al., 1999; Rudolph et al., 1999). Conversely, telomerase reactivation by TERT overexpression could reverse tissue degeneration in aged telomerase-deficient mice (Jaskelioff et al., 2011). Upregulation of hTERT and increased telomerase activity also improved the proliferative and colony-forming ability of hESCs by modulating the cell cycle dynamics (Yang et al., 2008). TERT-overexpressing hESCs displayed advantages in growth potential and stress resistance, and enhanced differentiation toward the hematopoietic lineage (Armstrong et al., 2005). Together, these findings provide a strong connection between telomerase status and stem cell pluripotency.

Given the importance of telomere maintenance in PSCs, the factors that can regulate telomerase expression, recruitment and activity are also expected to play a significant part in PSC biology. Several studies showed that pluripotency transcription regulators, also known as the four Yamanaka factors (OCT4, SOX2, KLF4 and C-MYC), could activate telomerase genes during reprogramming. For example, OCT3/4 and NANOG could bind to the TERC promoter and activate TERC transcription (Agarwal et al., 2010). Additionally, KLF4 was found to specifically and directly bind to the TERT proximal promoter and activate TERT expression in ESCs and iPSCs (Wong et al., 2010a; Hoffmeyer et al., 2012; Wang et al., 2012). KLF4 knockdown in human ESCs resulted in TERT expression downregulation and ESC differentiation, whereas TERT overexpression could rescue these phenotypes (Wong et al., 2010a). The factors that are required for telomerase RNA transcription and maturation should also play a central role in PSC maintenance. We recently found that TOE1 acts as a 3′ exonuclease for TERC/hTR processing and telomere maintenance (Deng et al., 2019). Future studies of TOE1 in PSCs will provide more understanding of the link between telomerase and pluripotency.

## TELOMERE HISTONES and EPIGENETIC MODIFICTIONS in PSCS

Previous studies have pointed the fundamental roles of chromatin epigenetic status in stem cell pluripotency maintenance (Meshorer and Misteli, 2006; Santos et al., 2010; Pfaff et al., 2013; Kobayashi and Kikyo, 2015; Ikeda et al., 2017). The undifferentiated stem cells contain a more open and active chromatin state when compared to the differentiated somatic cells. The differentiation process is always accompanied by a global change in chromatin histone modifications, including changes in active (acetylated H3K9 and H3K4me3) and repressive (H3K9me3 and H3K27me3) chromatin markers. Nuclear reprogramming also involves a large-scale resetting of chromatin structure and epigenetic status, which leads to a more open chromatin state. Despite it has been shown that chromatin structure could impact telomere maintenance in cancer cells, little is known of how chromatin structure affects telomere maintenance in pluripotent stem cells. Previous works have reported that ES cells and iPS cells contain less repressive telomeric chromatin when compared with the differentiated cells (Marion et al., 2009; Wong et al., 2009). mouse iPS reprogramming by retrovial transduction of pluripotency fators also results in a dramatic increase in telomere length to level functionally equivalent to those in mouse ES cells (Marion et al., 2009). We hypothesize that the chromatin status at telomere or subtelomere may has a direct impact on the telomere length and pluripotency maintenance in ES cells.

Besides core histones, the conserved histone variant H3.3 is also found to be associated with active/open chromatin. H3.3 can localize to telomeres in mESCs and embryonic germ cells, but not in non-pluripotent cells (Wong et al., 2009). During ESC differentiation, H3.3 levels at telomeres appeared to decease, which was companied by an increase of H4K20me3 and H3K9me3, both of which are markers of repressive heterochromatin (Marion et al., 2009). It is possible that H3.3 loading may prevent trimethylation of H4K20 and H3K9 and promote telomere elongation in pluripotent cells. These findings propose that H3.3 plays a key role in controlling telomere chromatin status and confers the capacity to maintain telomere length in ES cells. The relatively open telomere chromatin becomes more accessible to regulators such as telomerase or factors that mediate homologous recombination, which can both facilitate telomere elongation.

**Figure 2:**
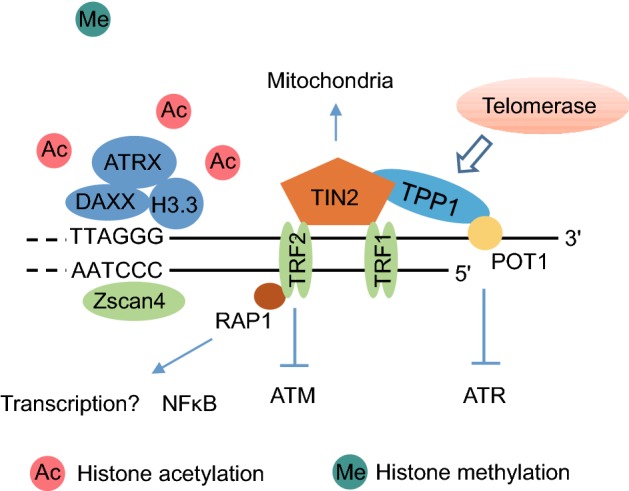
**Organization and molecular functions of the telomere binding proteins and histone modifications at hPSC telomeres**. Telomeric DNA is protected from DNA damage response by the shelterin/telosome proteins: the double-stranded telomeric DNA binding factors TRF1 and TRF2, the single-stranded telomeric DNA binding factor POT1, the organizing factors TIN2 and TPP1, and the TRF2-binding factor RAP1. Telomerase is recruited by the shelterin complex to elongate telomeres. Some extra-telomere roles of shelterin/telosome proteins are also shown as indicated. hPSCs show more open telomeric chromatin status, including more histone acetylation and less histone methylation. ATRX, DAXX and H3.3 work as a complex to bind to telomere and regulate telomere epigenetic status in hPSCs. Zscan4 localizes to telomeres and promote telomere elongation and epigenetic regulation in hPSCs

H3.3 is deposited on telomeres and pericentric heterochromatin by the ATRX/DAXX chaperone complex (Lewis et al., 2010; Wong et al., 2010b) (Fig. 2). ATRX is a member of the SNF2 family of helicase/ATPases that contributes to chromatin remodeling and repression. It has been shown that ATRX plays a unique function in regulation of telomere chromatin integrity in pluripotent mouse embryonic stem cells, but not in differentiated cells (Wong et al., 2010b). ATRX interacts with H3.3 and is required for the recruitment of HP1 at telomeres in pluripotent stem cells (Wong et al., 2010b). However, the level of ATRX at telomere is decreased during ES cell differentiation, suggesting an essential role of ATRX as a key regulator of telomere chromatin in pluripotency stem cells (Wong et al., 2010b). Moreover, ATRX and H3.3 colocalize with telomeric DNA at PML bodies, which serve as platforms for the maintenance of telomeric chromatin integrity in ESCs (Chang et al., 2013). These telomere-associated PML bodies (APBs) is associated with the pluripotent of mESCs, and is gradually reduced during ESC differentiation. Future studies are needed to define the functional link between ATRX and novel pathways involved in the regulation of telomeric chromatin status, telomere length homeostasis and pluripotency maintenance.

PSCs can tolerate global DNA hypomethylation. Our recent study showed that genome-wide demethylation promotes co-enrichment of DAXX and ATRX at tandem repetitive elements, including telomeres and subtelomeres, in mESCs and during early embryogenesis(He et al., 2015). The DAXX/ATRX complex is required for transcriptional repression and protection of repetitive elements in mESCs during DNA demethylation(He et al., 2015). Loss of DAXX or ATRX resulted in an increased IAP transcription in DNMT knockout cells. In addition, depletion of DAXX or ATRX in DNMT knockout cells, but not wild-type mES cells, exhibited increased fragile telomeres and dysregulated telomere length control. We also found that DAXX played a central role for DAXX/ATRX complex recruitment to telomere/subtelomere regions, and might involve in SUV39H1 recruitment and H3K9 trimethylation (He et al., 2015). Thus, the DAXX/ATRX complex is essential for repetitive sequences repression and protection in response to global DNA hypomethylation in mESCs and during early embryogenesis. Further investigation of the regulation of DAXX and ATRX during embryogenesis and ES cell differentiation should shed light on the mechanisms of pluripotency maintenance and embryo development.

### Zscan4 IN PSCS

Zscan4 is specifically expressed in late 2-cell stage embryos and ESCs, and inactivated following ESC differentiation (Falco et al., 2007). It was found that Zscan4 could target to telomeres and promote telomere elongation by homologous recombination (HR) in ESCs (Zalzman et al., 2010) (Fig. 2). During early embryonic development, T-SCE and HR are required for telomere elongation (Liu et al., 2007). Perhaps Zscan4 promotes HR-mediated lengthening of telomeres in early embryos. However, the exact mechanisms underlying Zscan4-mediated recombination regulation remain to be determined. Zscan4 has also been found to contribute to telomere elongation and reprogramming during SCNT and iPSC generation, although Zscan4 overexpression was only required for the first few days of iPSC formation (Hirata et al., 2012). Further studies have demonstrated a role of Zscan4 in protecting genome stability during reprogramming and improving the quality of iPSCs (Jiang et al., 2013). Previous studies showed that epigenetic status might play a critical role in Zscan4 regulation. Histone hyperacetylation activates Zscan4 expression, whereas histone hypoacetylation represses (Dan et al., 2015). DNA hypermethylation induced by Tet1/2 double-knockout (DKO) or Tet1/2/3 triple-knockout (TKO) upregulates Zscan4, which may compensate for telomere shortening (Lu et al., 2014). In addition, TBX3, a pluripotency maker required for self-renewal of ESCs and iPSCs (Han et al., 2010; Lu et al., 2011), activates Zscan4 gene expression and elongates telomeres in mESCs via indirect effects on DNA methylation (Dan et al., 2013). Again, more work is needed (e.g., single-cell sequencing) to understand how Zscan4 is regulated during stem cell differentiation and embryo development.

## THE SHELTERIN/TELOSOME COMPLEX IN PSCS

The six-subunit shelterin/telosome complex comprises of three DNA-binding proteins (TRF1, TRF2, and POT1) and three proteins with no demonstrable DNA-binding activities (TPP1, TIN2, and RAP1) (Xin et al., 2008; de Lange, 2018) (Fig. 2). These six core telomere-associated proteins regulate telomerase access to telomeres, protect telomere ends from DNA damage response, and ensure proper telomere maintenance by recruiting other factors (Xin et al., 2007; Abreu et al., 2010; Nandakumar et al., 2012; Sexton et al., 2012; Zhong et al., 2012). Through the concerted efforts of telomerase and telomere-binding proteins, cells are able to counteract the end replication problem in both somatic cells and PSCs. Further, extra-telomeric functions of shelterin/telosome proteins in pluripotency maintenance have also begun to be appreciated.

### TRF1

The telomere repeat factor 1 or TRF1 binds to the double-stranded region of telomeres (Court et al., 2005). TRF1 was found to control telomere length negatively and promote telomere replication (van Steensel and de Lange, 1997; Sfeir et al., 2009). During *in vitro* derivation of ES-cell lines from the inner cell mass (ICM), increased TRF1 expression was observed before the onset of telomere elongation (Varela et al., 2011). Highly upregulated expression of TRF1 was also found in ESCs and iPSCs, independent of of the length status of telomeres (Boue et al., 2010; Schneider et al., 2013; Marion et al., 2017). These findings suggest that TRF1 may serve as a pluripotency marker. Moreover, TRF1 is necessary for both induction and maintenance of pluripotency. Knocking out TRF1 reduced *in vivo* reprogramming efficiency. Interestingly, TRF1 is also a direct transcriptional target of Oct3/4 (Schneider et al., 2013). It suggests that high level of TRF1 in stem cells is conducive to telomere replication and genome stability.

### TRF2

Like TRF1, the telomere repeat factor 2 or TRF2 also binds to double-stranded telomere DNA (Bilaud et al., 1997) . TRF2-binding is thought to facilitate T-loop formation (Stansel et al., 2001). TRF2 has been shown to suppress ATM signaling activation and prevent non-homologous end joining (NHEJ) and homology-directed repair (HDR) at telomeres (Celli et al., 2006; Bae and Baumann, 2007; Denchi and de Lange, 2007) (Fig. 2). Loss of TRF2 can lead to telomere uncapping and activation of DNA damage response at telomeres (van Steensel et al., 1998; Kim et al., 2017). Interestingly, TRF2 deletion in alveolar stem cells was found to limit their self-renewal and differentiation (Alder et al., 2015). In comparison, TRF2-overexpresion in mouse induces epidermal stem cells dysfunction with critically short telomeres. Meanwhile, p53 deletion could rescues severe phenotypes in TRF2-overexpression mice, suggesting that DNA damage response involve in epidermal stem cells dysfunction (Stout and Blasco, 2009). Notably, TRF2 also poses an essential role in controlling neural stem cell fate, which is independent of telomere function. It has been reported that TRF2 is required for embryonic and adult neurogenesis (Ovando-Roche et al., 2014; Lobanova et al., 2017), but dosen’t play a role in terminally differentiated neurons. TRF-S, one splicing type of TRF2 in neural stem cells, prefer to bind mRNA but not telomeric DNA. The findings suggest a pivotal role for TRF2-S in axonal mRNA localization that enhances axon outgrowth and neurotransmitter release (Zhang et al., 2008; Zhang et al., 2015a; Grammatikakis et al., 2016). Beyond All That, TRF2 could be found at genomic DNA to regulate genes transcription and heterochromatin maintenance (El Mai et al., 2014; Mendez-Bermudez et al., 2018). Partial deletion of TRF2 increases the radiosensitivity of human mesenchymal stem cells (Orun et al., 2016; Serakinci et al., 2018). In all, TRF2 plays essential roles in stem cell state controlling, and it may have extra-telomere functions in this regulation. However, the functions of TRF2 independent of telomere are not clear in pluripotent stem cells, and more studies are needed to indicate its possible role in pluripotency maintenance.

### TPP1 and POT1

Of the six core telomere proteins, only POT1 has the ability to specifically bind the G-rich single-stranded telomere overhang (Baumann and Cech, 2001). It is required for suppressing ATR-mediated DNA damage response (Churikov et al., 2006; Denchi and de Lange, 2007) (Fig. 2). Most vertebrates, including humans, possess a single POT1 gene. But there are two POT1 genes (POT1a and POT1b) in rodent (Hockemeyer et al., 2006). Knockdown POT1a or knockout POT1b in mice result in increased telomeric DNA damage, and lead to apoptosis of hematopoietic stem cells (Wang et al., 2011; Hosokawa et al., 2017). TPP1 was identified as interacting protein of POT1 in three independent research group (Houghtaling et al., 2004; Liu et al., 2004b; Ye et al., 2004b). The mutation of TPP1 would lead to adrenocortical dysplasia, which is a disease with developmental defects in organs derived from the urogenital ridge (Keegan et al., 2005). TPP1 functions as a molecular mini-hub at the end of telomeres, by interacting with POT1, TIN2, and TERT (O’Connor et al., 2006; Xin et al., 2007). The TPP1-POT1 heterodimer functions analogously to ciliate TEBP to facilitate telomerase recruitment, while TPP1-TIN2 interaction helps bridge the double- and single-stranded DNA binding activities of the shelterin/telosome (Wang et al., 2007; Xin et al., 2007). TPP1 depletion in cancer cells caused DNA damage response at telomeres and led to telomere dysfunction (Guo et al., 2007; Xin et al., 2007). Knockdown SIRT1 reduce TPP1 expression in young mesenchymal stem cells (MSCs), leading telomeric DNA damage and cell senescence (Chen et al., 2014). TEL patch mutation of TPP1 suppresses telomerase recruitment and induce cell cycle arrest or apoptosis in certain cancer cells (Nakashima et al., 2013). Indeed, abrogation of TPP1 also abolished telomere elongation in human embryonic stem cells and mouse embryonic fibroblast (MEF) reprogramming, supporting a role of TPP1 in maintenance of PSCs (Tejera et al., 2010; Sexton et al., 2014).

### TIN2

TIN2 (TRF1-interacting nuclear factor 2) was initially discovered as a protein that could interact with TRF1 (Kim et al., 1999). It has since been shown to function as a central component of the shelterin/telosome complex. TIN2 can interact with both TRF1 and TRF2 on the one hand, and TPP1 on the other, thereby bringing together the double- and single-stranded DNA-binding activities within the complex for telomere maintenance (Kim et al., 1999; Liu et al., 2004a; Ye et al., 2004a; O’Connor et al., 2006; Chen et al., 2008; Kim et al., 2009; Takai et al., 2011). The interaction between TIN2 and TPP1 also important for telomerase recruitment to telomere (Abreu et al., 2010; Yang et al., 2011). Knocking out (KO) TIN2 in mice is embryonically lethal and CRISPR-mediated TIN2 KO in human cells also led to cell death (Chiang et al., 2004; Kim et al., 2017). These observations support extra-telomeric functions of TIN2. Indeed, TIN2 has been shown to also localize to the mitochondria and regulates oxidative phosphorylation (Chen et al., 2012). Additionally, mutations of TIN2 have been identified in patients with dyskeratosis congenita (DC), a syndrome characterized by bone marrow failure and somatic stem cell dysfunction in multiple organs, such as the epidermis and the hematopoietic system. More work is needed to understand the molecular and cellular consequences of TIN2 mutations to pluripotent stem cells.

### RAP1

Repressor/activator protein 1 or RAP1 localizes to telomere via its interaction with TRF2 (Li et al., 2000; Chen et al., 2011). The RAP1-TRF2 heterodimer protects telomeres from inappropriate HDR and NHEJ activation (Bae and Baumann, 2007; Sfeir et al., 2010; Rai et al., 2016). RAP1 deficiency can result in telomere shortening in human cells (O’Connor et al., 2004). While RAP1 KO in mice did not lead to embryonic lethality, in addition to shortened telomeres, hyperpigmentation and increased DNA damage activation could also be observed in adult RAP1 KO mice (Martinez et al., 2010). Mammalian Rap1 associates with DNA through its interaction with TRF2, including telomere and extra-telomere. Extra-telomeric RAP1-binding sites have been found in the vicinity of genes, suggesting a role for RAP1 in transcriptional control (Martinez et al., 2010; Yang et al., 2011; Martinez et al., 2013). Not all RAP1 proteins are in the nucleus. Cytoplasmic Rap1 is an inhibitor kinase (IKK) adaptor that controls NFκB pathway (Teo et al., 2010). Suppression of NFκB pathway through knockdown RAP1 is benefit for atherosclerotic lesions and myocardial infarction (Cai et al., 2015; Zhang et al., 2015b). To further investigate the telomeric and extra-telomeric functions of RAP1 should help shed more light on its possible role in pluripotency maintenance.

## CONCLUSIONS AND PERSPECTIVES

Multiple links have been established between telomere homeostasis and stem cell pluripotency maintenance. Sufficient telomere length is critical for unlimited self-renewal of PSCs. Telomere length and integrity may serve as general signatures to evaluate the quantity of ESCs and iPSCs. Longer telomeres would be advantageous for human ESCs and iPSCs in the application of PSCs in regenerative medicine. However, excessive telomere elongation heightens sensitivity of hESCs to replication stress at overly long telomeres, and causes the formation of C-circles (Rivera et al., 2017). Thus, the tight control of telomere length homeostasis is essential for stem cell status maintenance. In addition, insight into telomere length regulation in PSCs may have implications in understanding aging and tumorigenesis and lead to discoveries of novel anti-cancer treatment.

Telomere length maintenance in ESCs and iPSCs is regulated by telomere-binding modulators and epigenetic modifications. Additionally, higher level of the shelterin/telosome component TRF1 also correlates with higher pluripotency of stem cells. Numerous studies have elucidated the roles of these telomere-binding proteins in telomere homeostasis. In the future, more focused effort should be made to delineate how stem cell pluripotency is regulated by telomere-binding modulators. Epigenetic changes at telomeres are acquired during reprogramming and ESC differentiation, these epigenetic modifications may provide a more flexible and efficient system to control of telomere length regulation in pluripotent cells. Future studies into how these telomeric chromatin status is regulated and contributes to the telomere homeostasis would provide insights to understand the mechanism of pluripotency maintenance and aging.

Mutations in telomere proteins have been identified in many human diseases including DC. Our previous systematic analysis of human shelterin/telosome knockout cells has revealed functional differences between human and mouse telomeric proteins in telomere length homeostasis and end-protection (Kim et al., 2017). In addition, extra-telomere functions of telomere proteins highlight an essential link between telomeres and stem cell pluripotency. Higher level of TRF1 correlates with higher pluripotency of stem cells. TRF2-RAP1 could be found at genomic DNA to regulate genes transcription and heterochromatin maintenance. TIN2 has been shown to localize to the mitochondria and regulate oxidative phosphorylation. The extra-telomere functions of shelterin/telosome components will be found in pluripotency maintenance. *In vivo* studies will be necessary to fully understand the molecular functions of telomere-binding proteins in somatic stem cells and tissue development.

The role of telomere binding proteins in the stem cell self-renewal and pluripotency maintenance has been well discussed. However, the functions of these binding proteins in stem cell differentiation are still rarely studied. During the differentiation process, the gradually shorter telomeres may release some of the telomere binding proteins from telomeres or recruit some new factors to telomeres, which may affect the process of differentiation. Future studies should focus on the specific role of these telomere binding modulators in stem cell differentiation. In addition, telomere shortening or uncapping in stem cells leads to cell senescence and aging. However, previous studies usually focus on cellular level, but rarely mention individual level. What are the differences of telomere binding proteins in stem cells between young and elder individuals? How these differences contribute to individual stem cell therapy? Further studies are needed.
